# Skeletal pathologies track body plan evolution in ichthyosaurs

**DOI:** 10.1038/s41598-020-61070-7

**Published:** 2020-03-06

**Authors:** Judith M. Pardo-Pérez, Benjamin P. Kear, Erin E. Maxwell

**Affiliations:** 1 Staatliches Museum für Naturkunde Stuttgart, Rosenstein 1, 70191 Stuttgart, Germany; 2 Vicerrectoría de Investigación y Postgrado, Universidad de Magallanes, Avenida Bulnes, 01855 Punta Arenas, Chile; 3 Centro de Investigación GAIA-Antártica, Universidad de Magallanes, Avenida Bulnes, 01855 Punta Arenas, Chile; 4 Museum of Evolution, Uppsala University, Norbyvägen 16, SE-752 36 Uppsala, Sweden

**Keywords:** Palaeontology, Palaeontology, Evolution, Solid Earth sciences, Diseases, Trauma

## Abstract

Changing predator-prey interactions during the Mesozoic Marine Revolution (MMR) profoundly altered the trajectory of marine tetrapod evolution. Here, we assess potential signatures of this landmark transition through the fossil record of skeletal pathologies in ichthyosaurs — iconic marine reptiles that developed increasingly ‘fish-like’ body plans over time. We surveyed a stratigraphically constrained sample of 200 Middle Triassic ichthyosaur specimens and compared the type, distribution and prevalence of pathologies with an approximately equivalent assemblage of Early Jurassic age. Overall, skeletal pathologies were equally prevalent in these groups, and most often manifested in species >4 m long. However, pathological bones were found to be concentrated in the hind limbs and tail of Triassic ichthyosaurs, whereas the jaws, forelimbs, and ribcage were preferentially affected in Jurassic taxa. We posit that the occurrence of ankylosed zygapophyses in the caudal peak of Triassic ichthyosaurs could represent a functional by-product of their primitive ‘eel-like’ swimming. Conversely, increased instances of broken ribs in Jurassic ichthyosaurs may infer ramming or tail strike behaviours that characterise morphologically ‘fish-like’ marine tetrapods, such as modern toothed whales. Different categories of skeletal pathologies thus evidently reflect structural modifications in the ichthyosaur body plan, and indirectly coincide with ecological turnover during the MMR.

## Introduction

The Mesozoic Marine Revolution (MMR) was coined to describe the reorganization of benthic ecosystems in response to escalating predation pressure and the rise of modern marine faunas^1,2^. A key aspect of this biotic watershed was the increase in diversity and abundance of pelagic marine reptiles^3^, whose rich fossil record evinces corresponding changes in ecology and evolution. Ichthyosaurs were one of the most successful of these groups, and constituted globally distributed pelagic predators that occupied mid- to high trophic levels from the Early Triassic to the Late Cretaceous^4^. Direct evidence of predator-prey, and intraspecific interactions involving ichthyosaurs can be assessed using preserved examples of skeletal pathologies. These traces reveal not only instances of injury and disease, but can be used to infer feeding and intraspecific behaviours, as well as locomotory functions and life history^5–7^.

Skeletal pathologies have been well-documented in ichthyosaurs of Jurassic and Cretaceous age^8–11^, but to date, relatively few cases have been reported from Triassic ichthyosaur specimens^8^. This phenomenon has been attributed to escalating predation pressures, and the subsequent reshaping of marine vertebrate ecosystems during the MMR^11^. Incongruously, however, recent surveys have shown that the frequency of diagnosable ichthyosaur bone pathologies is dramatically under-evaluated^10^. Consequently, to test whether palaeoecological changes during the MMR actually exerted a measurable impact on the occurrence of skeletal pathologies in extinct marine tetrapods, we quantified a stratigraphically constrained sample of ichthyosaur fossils from the Middle Triassic Besano Formation of the Swiss-Italian Alps, and compared the type, distribution and prevalence of recognisable bone traumas and disease with an ecologically analogous assemblage recovered from the Lower Jurassic Posidonienschiefer Formation of southwestern Germany^10^. We treated all non-congenital skeletal damage incurred during the lifetime of the animal as pathological, including traumatic injuries, infection, post-natal articular ankyloses, and articular disease; this follows the classification scheme of Pardo-Pérez *et al*.^9^. These approaches hypothesize that the ubiquitous constraints of an aquatic lifestyle will leave commensurate characteristics of pathological bone modification in fossil marine tetrapod assemblages given proportionate preservation, stratigraphical constraints and geographical representation. Ultimately, we aim to determine whether an analysis of skeletal pathologies in representative samples of Triassic versus Jurassic ichthyosaur skeletons can meaningfully track the large-scale changes in marine reptile ecosystems that occurred in conjunction with the MMR^11^.

## Materials and Methods

### Institutional abbreviations

BES SC: Museo Civico di Storia Naturale di Milano, Italy. SMNS: Staatliches Museum für Naturkunde Stuttgart, Germany. GPIT: Palaeontological Collection of Tübingen University, Germany. PIMUZ: Paläontologisches Institute und Museum Universität Zürich, Switzerland.

### Experimental assemblage characterisation

The Besano Formation crops out along the Swiss-Italian border and preserves a diverse marine vertebrate fauna. Ichthyosaurs constitute some of the most common reptile fossils, and derive primarily from the upper Anisian (lower Middle Triassic) section of the unit^12^. We surveyed examples of skeletal pathologies in 200 Besano Formation ichthyosaur specimens housed in museum collections across Europe (Supplementary Table S3). Taxonomically, the remains are referred to the cymbospondylid *Cymbospondylus* (*n* = 3), the shastasaurids *Besanosaurus*, *Wimanius* and *Mikadocephalus* (*n* = 7), and the mixosaurids *Mixosaurus* and *Phalarodon* (*n* = 190). These taxa also correspond to discrete body size categories: *Cymbospondylus* is estimated at 6–10 m in maximum length^13^; *Besanosaurus*, *Wimanius* and *Mikadocephalus* range from 4–8 m in maximum length^14^; and *Mixosaurus* and *Phalarodon* both are <2 m long. Reconstructed trophic relationships suggest that *Cymbospondylus* was an apex predator^15^, whereas the smaller-bodied ichthyosaurs were all likely middle trophic-level feeders.

**Figure 1:**
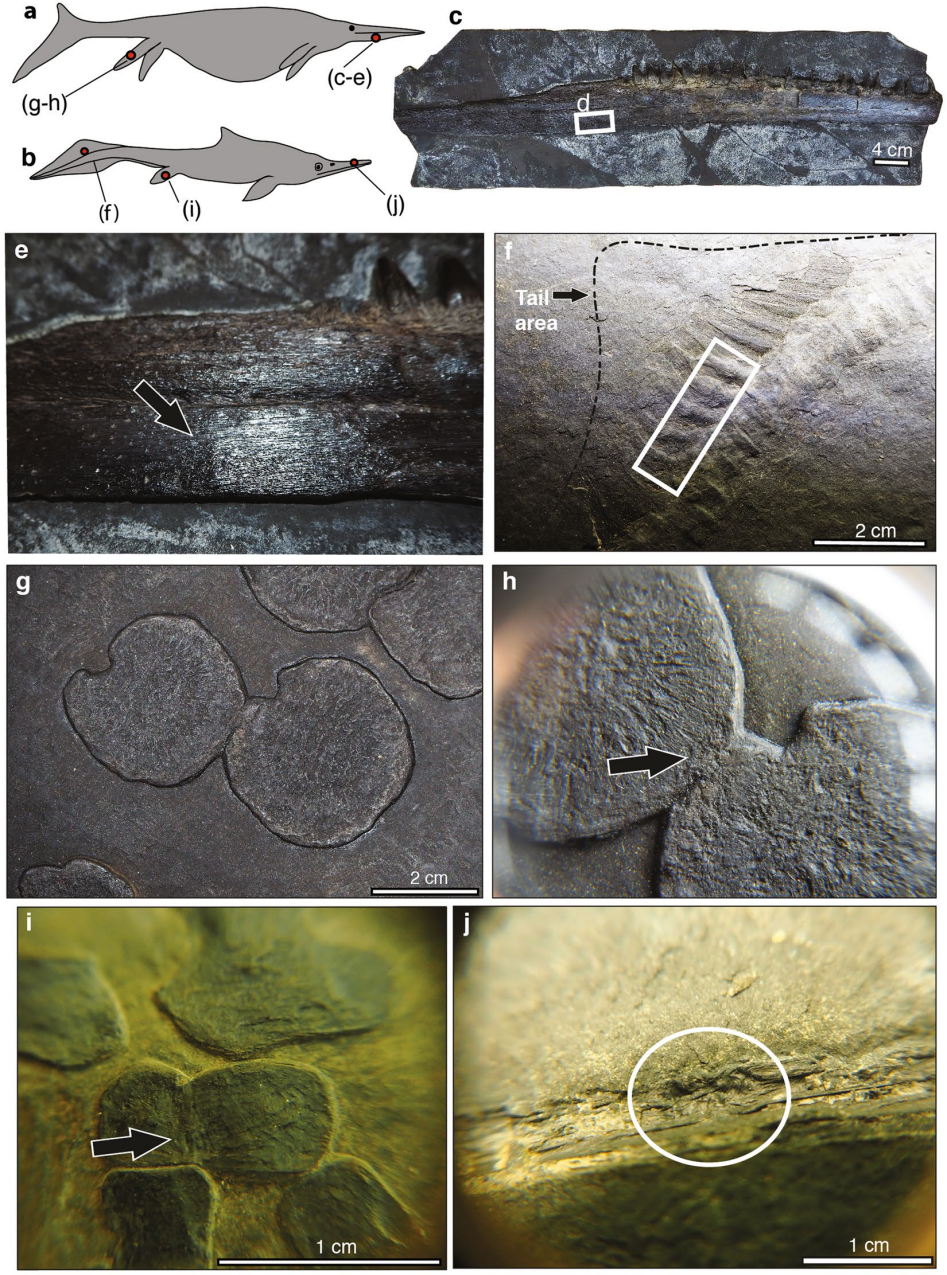
Examples of pathological bone modifications detected in ichthyosaur skeletons from the Middle Triassic Besano Formation of the Swiss-Italian Alps. (**a**,**b**) Anatomical representation of a shastasaurid (**a**) and a mixosaurid ichthyosaur (**b**) showing the affected areas. (**c**–**e**). Healed trauma in the dentary of an indeterminate shastasaurid (PIMUZ T 39). (**f**) Articular disease affecting the zygopophyseal region in the apical region of the tail of *Mixosaurus* (BES SC, unregistered specimen). (**g**,**h**) Ankylosis in the hind limb metapodial elements of *Besanosaurus* (BES SC 999). (**i**) Ankylosis of the proximal tarsals in the hind limb of *Mixosaurus* (PIMUZ T 2417). (**j**) Healed trauma with evidence of bone remodeling in the premaxilla of *Mixosaurus* (PIMUZ T 2140).

We categorised all non-congenital damage observed on the Besano Formation ichthyosaur specimens as pathological, and included healed traumatic injuries, infection, post-natal articular ankyloses and articular disease (see examples in Fig. 1). These categories were then grouped according to the affected skeletal region^8,16^: (1) skull; (2) ribs and gastralia; (3) vertebral column; (4) pectoral girdle and forelimb; and (5) pelvic girdle and hind limb.

Ontogenetic effects were assessed in only the most abundant and skeletally complete clade — Mixosauridae (*Mixosaurus* and *Phalarodon*). All specimens for which size could be estimated (*n* = 104) were grouped into two size classes: ‘small’, interpreted as ‘juveniles’ and represented by individuals with mandibular lengths <200 mm, humeral lengths <24 mm, and femoral lengths <14 mm; and ‘large’, defined as ‘adult’ individuals with mandibular lengths between 210–420 mm, humeral lengths from 25–50 mm, and femoral lengths of 15–30 mm. Anatomical region was included as a covariate to control for potential differences in completeness between these size classes. We analysed our resulting dataset using binomial logistic regressions (BLRs) performed with the software platform R^17^. This allowed for selection of multiple categorical predictor variables (e.g. taxon + anatomical region), as well as a binary response variable (pathology: present/absent).

To test for differences in the type and prevalence of skeletal pathologies between Triassic and Jurassic ichthyosaur assemblages, we compared the Besano Formation dataset (Supplementary Table S3) to a Lower Jurassic dataset of ichthyosaurs from Posidonienschiefer Formation of southwestern Germany^10^. We first qualitatively compared examples of pathological bone modifications across all taxa in both these experimental assemblages, and then quantitatively focused on Mixosauridae versus the parvipelvian *Stenopterygius* as the most abundant genus-level representatives. We decided to apply the broad clade designation Mixosauridae for this analysis because *Mixosaurus* and *Phalarodon* cannot be taxonomically differentiated without representative cranial material.

Both *Stenopterygius* and mixosaurids are amongst the smallest-bodied ichthyosaurs in their respective ecosystems, and are known to have primarily preyed upon cephalopods as adults^18,19^. Because pathologies were not observed on the ribs and gastralia of our mixosaurid specimens, we were unable to perform our BLRs with ‘0’ values. Consequently, we added a single simulated observation for this category to be able to proceed with our analysis.

## Results

### Types of skeletal pathologies

We found that traumatic injuries and articular ankyloses were equally prevalent in all taxa, size, and trophic-levels classes within our Besano Formation ichthyosaur assemblage (3%; 6/200; see Fig. 1, Supplementary Table S1). Traumatic injuries were recognized in the jaws (premaxilla and dentary) of both small and large-bodied taxa, and in the femur of one individual of *Mixosaurus* (PIMUZ T 2420, *Mixosaurus cornalianus*). A post-traumatic infection was detected in the pectoral girdle (left clavicle, scapula and coracoid) of the holotype of *Cymbospondylus buchseri* (PIMUZ T 4351). We further observed that ankyloses tended to occur in the distal zeugopodial elements of the fore- and hind limbs in *Mixosaurus* (PIMUZ T 2417, PIMUZ T 2412 and PIMUZ T 2408). Articular disease was the least often identified pathological condition (1%; 2/200), and was observed on the neural spine bases of the caudal peak region of *Mixosaurus* remains from the BES SC collection (BES SC 1000 and BES SC unregistered specimen) (*sensu*^20^).

### Distribution of skeletal pathologies

The most frequently injured and/or diseased skeletal regions encountered in the Besano Formation ichthyosaur fossils were the pelvic girdle and hind limb (7%; 4/57), followed by the skull (3%; 4/145), pectoral girdle and forelimb (2%; 2/102), and the vertebral column (2%; 3/133). No pathologies were observed on the ribs or gastralia, and no substantial differences were detected between different skeletal regions in our mixosaurid sample (see Supplementary Table S2).

### Taxonomic, size and trophic-level variation

The highest prevalence of skeletal pathologies in the Besano Formation ichthyosaurs occurred in the apex predator taxon *Cymbospondylus* (67%; 2/3), followed by the middle trophic-level shastasaurids *Besanosaurus*, *Wimanius* and *Mikadocephalus* (29%; 2/7), and the mixosaurids *Mixosaurus* and *Phalarodon*, which exhibited the least number of identifiable pathologies (5%; 9/190).

### Ontogenetic variation

Based on our ontogenetic proxy for mixosaurids, we found no substantial difference in the prevalence of skeletal pathologies between ‘adult’ (7%; 3/42) and ‘juvenile’ size classes (6%; 4/62).

### Variation between chronostratigraphically constrained assemblages

**Figure 2:**
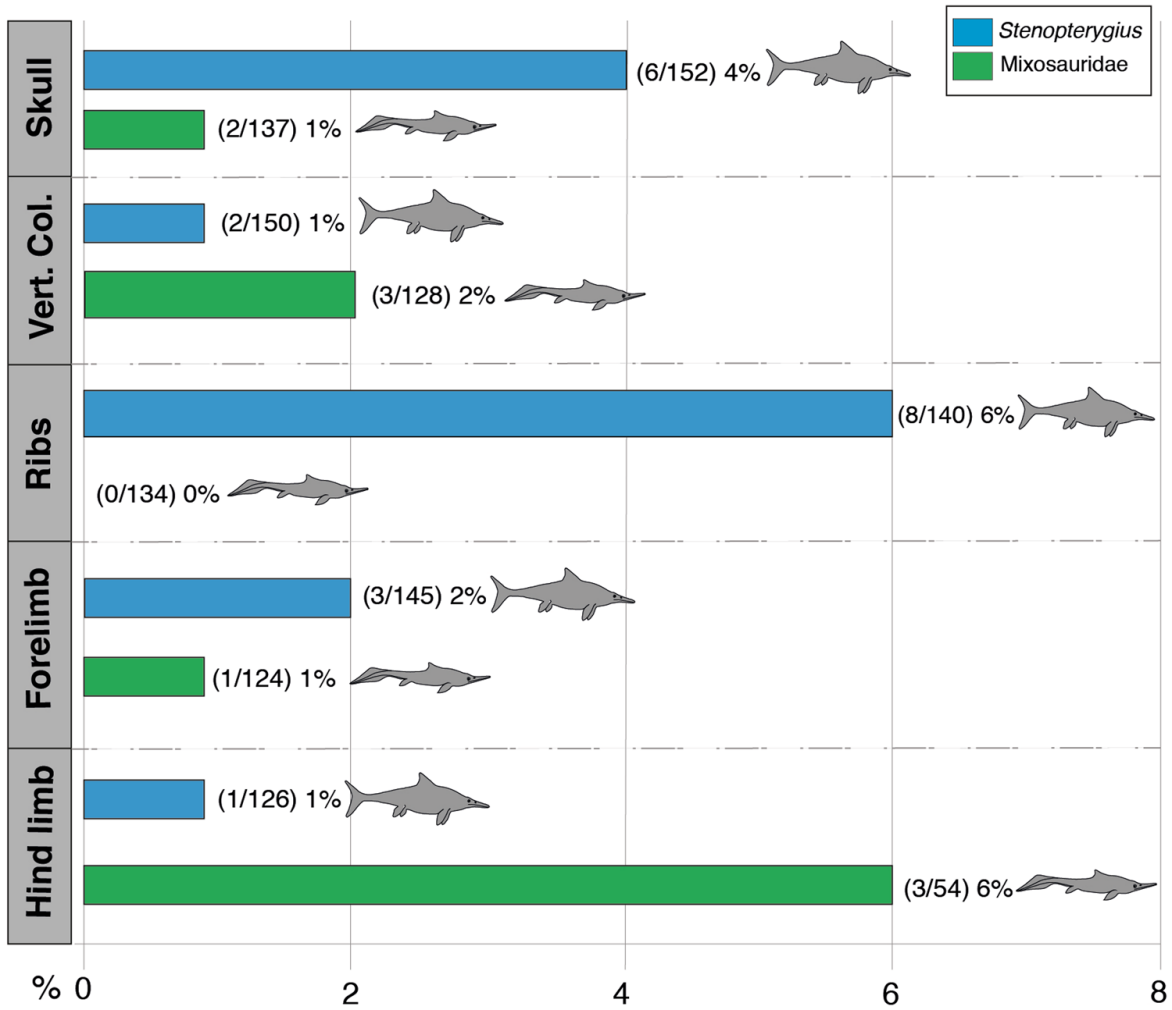
Graphic illustrating the comparative prevalence and distribution of bone pathologies in the most abundant and skeletally complete Besano Formation (Mixosauridae) versus Posidonienschiefer Formation (*Stenopterygius*) ichthyosaur taxa.

Qualitative comparison of skeletal pathologies across all of the identified taxa in the Besano and Posidonienschiefer Formation ichthyosaur assemblages indicated that those representative genera with maximum estimated body lengths exceeding 4 m (*Cymbospondylus*, *Besanosaurus*, *Wimanius* and *Mikadocephalus* versus *Temnodontosaurus* and *Eurhinosaurus*, respectively) were more likely to exhibit evidence of injuries and disease than the smaller-bodied coeval forms (*Mixosaurus* and *Phalarodon* versus *Stenopterygius* and *Hauffiopteryx*, respectively). Furthermore, we detected no substantial difference in the overall prevalence of skeletal pathologies between the most abundant taxa (Mixosauridae versus *Stenopterygius;* Fig. 2; Supplementary Table S2); however, the distribution of pathologies varied considerably between these taxa. *Mixosaurus* showed the highest prevalence of pathologies in the pelvic girdle and hind limb (6%; 3/54), as opposed to the vertebral column (2%; 3/128), pectoral girdle and forelimb (1%; 1/124), and skull (1%; 2/137). No damage was observed on the ribs and gastralia, which differs markedly from the recorded specimens of *Stenopterygius* where this region was most often injured (6%; 8/140)^10^. Likewise, the skulls (4%; 6/152) of *Stenopterygius* more commonly suffered pathological damage, whereas the pectoral girdle and forelimbs (2%; 3/145), vertebral column (1%; 2/150) and pelvic girdle and hind limbs (1%; 1/126) manifested fewer pathological skeletal modifications.

## Discussion

Our comprehensive survey of ichthyosaur skeletons from the Middle Triassic Besano Formation reveals a range and prevalence of diagnosable pathologies that is approximately equivalent to those reported from stratigraphically younger deposits^10^. This result concurs with other recent studies, which assert that pathological bone modifications are, in general, under-reported^9,10^. We also counter suggestions that instances of injury and disease are rare in Triassic ichthyosaur remains^8,21^, and that this indicates a shift towards increased predation pressure influencing pelagic marine reptile communities across the Triassic–Jurassic interval^11^.

As an alternative, we demonstrate that the dominant ichthyosaur taxa bracketing this transition exhibit disparity in their skeletal regions being affected by pathological damage. For example, traumatic injuries to the ribs and gastralia are conspicuously absent in the Besano Formation specimens, whereas they occur much more frequently in post-Triassic ichthyosaur assemblages^22–28^. Such healed rib fractures are often interpreted as products of intraspecific aggression^9,10,29^, and like bite traces found on the jaws of *Cymbospondylus*, shastasaurids, and mixosaurids, probably resulted from aggressive encounters with conspecifics, as has been hypothesized for other ichthyosaurs^9,30^. Extant odontocete cetaceans also employ biting, as well as body slamming, high-velocity ramming, and tail strikes during aggressive interactions^22–28^. These behaviours can result in broken ribs or even death, and are enabled by the powerful tail fin, which concentrates axial displacement in the caudal peduncle and is characteristic of a fusiform (=axially tapered, or spindle-shaped) body plan. In contrast, Triassic ichthyosaurs typically relied on ‘eel-like’ (=anguilliform) locomotion, in which the torso and caudal region both needed to be flexible for undulatory propulsion. Moreover, broken ribs would have been especially debilitating for an anguilliform swimmer, which presumably integrated substantial torso flexion to generate thrust. We therefore propose that the increased instances of rib injuries throughout the ichthyosaur fossil record may reflect progressive adaptation towards more fusiform body shapes, and the corresponding radiation of advanced parvipelvian, or classically ‘fish-like’ ichthyosaurs during the latest Triassic.

We additionally show that the prevalence of vertebral ankyloses is approximately equivalent between our Triassic and Jurassic ichthyosaur assemblages (Supplementary Table S2). However, the distribution of these pathological modifications differs substantially along the column, manifesting in the caudal peak zygapophyses of Triassic taxa (e.g., mixosaurids), as opposed to the presacral neural spines in Jurassic taxa (e.g., *Stenopterygius*)^10^. This pattern is strikingly similar to that observed amongst Cretaceous mosasaurine marine lizards, in which ankyloses in the caudal peak region seemingly formed as a result of mechanical stresses from undulatory swimming^31,32^. Other conditions, including infectious spondylitis and ligamentous ossifications, also cause ankylosis of the caudal vertebrae in many aquatic tetrapods^33–39^. Pointedly, though, we detected no traces of avascular necrosis in any of our Besano Formation ichthyosaurs, which is consistent with previous reports on other Triassic taxa^21^, and could indicate a preference for shallower water habitats and/or limited diving capabilities in the species that we examined.

Recent studies have shown that ichthyosaur skeletal pathologies can be intraspecifically correlated with body size as a measure sexual maturity: larger ‘adults’ displaying more numerous injuries and age-related conditions, including ankyloses and articular disease^10^ (conditions that typically coincide with osteological maturity in vertebrates^40,41^). Conversely, our data from the small-bodied mixosaurid ichthyosaurs indicated proportionate numbers of diagnosable pathological cases in both ‘adult’ and ‘juvenile’ size classes, with ‘juveniles’ exhibiting higher incidence of bone traumas and ankyloses. Unlike *Stenopterygius*, for which body size range at osteological and sexual maturity is known^42,43^, this information is not readily available for *Mixosaurus* or *Phalarodon*. The only *Mixosaurus* specimen in our sample incorporating directly associated embryonic remains^44^ has a mandibular length of 220 mm, and thus marginally exceeded our designated <200 mm ‘juvenile’ size class cut-off, implying that miss-identification of smaller ‘adults’ might be artificially normalising our results.

The distribution of limb bone ankyloses in our surveyed assemblage of mixosaurids was likewise atypical in being localized to the mesopodia, whereas both congenital and pathological ankyloses are usually dispersed throughout the distal limb elements in advanced parvipelvians, such as *Stenopterygius*^10,45^. Pathological limb bone ankyloses affect the joint surfaces, and are linked to locomotion^6,33,46–49^. However, in *Mixosaurus* and other primitive ichthyosaurs, articular contacts are restricted to the distal ends of the phalanges, with the leading and trailing edges instead retaining dense cortical bone. In contrast, the limb elements of parvipelvians are arranged in a tightly interlocking ‘pavement’ that maintains multiple articulations between adjacent digits, and thus could explain our observed increase in the prevalence of distal joint ankyloses. Notably, we detected an isolated case of joint ankylosis in the metatarsals of *Besanosaurus*; however, this fusion did not involve a region typically covered with cortical bone (Fig. 1d,e).

In conclusion, our study finds that skeletal pathologies are widespread throughout the ichthyosaur fossil record. However, their type, prevalence and distribution reveals the changing locomotory and behavioural constraints affecting different taxa through time. Skeletal pathologies can thus demonstrably track aspects of the adaptive transition to increasingly pelagic lifestyles, and provide valuable information about the biology of ichthyosaurs as extinct animals. On the other hand, our data do not obviously reflect any large-scale trophic rearrangements^11^. Consequently, successive changes in the body plan of Triassic versus Jurassic ichthyosaurs would appear to have exerted a more immediately measurable effect on the documented record of palaeopathologies than any more broadly impacting ecosystem-level perturbations (e.g., an increasing rate of predation over time). We therefore caution against drawing direct causal links with the MMR, which although undoubtedly influential in shaping modern oceanic biotas, at present, only constitutes a coincidental backdrop for the history of injury and disease in ancient marine reptiles.

## Supplementary information

Supplementary information 1.

Supplementary information 2.

## Data Availability

All data generated or analysed during this study are included in this published article (and its Supplementary Information Files).
