# Supplementary material for: Skeletal pathologies track body plan evolution in ichthyosaurs
Source: Sci Rep. 2020 Mar 6;10:4206. doi: 10.1038/s41598-020-61070-7 (PMC7060314; doi:10.1038/s41598-020-61070-7)
Supplement: Supplementary file 1 — Supplementary information 1. [file 41598_2020_61070_MOESM1_ESM.doc]

**Supplementary information from** “**Skeletal pathologies track body plan evolution in ichthyosaurs ”**

Judith M. Pardo-Pérez^1,2,3*^, Benjamin P. Kear^3^ and Erin E. Maxwell^1^

**SCIENTIFIC REPORTS**

**Supplementary Table S1.** Logistic regression with aggregated data. Independent variables: Size and anatomical region of mixosaurids (categorical predictors), pathological (binary response)

| **Size (cm)** | **Anatomical region** | **Pathological** | **Not** | **Total** | **%** |
| --- | --- | --- | --- | --- | --- |
| <20 (juvenile) | Skull | 1 | 51 | 52 | 2% |
| <20 (juvenile) | Ribs + Gastralia | 0 | 42 | 42 | 0% |
| <20 (juvenile) | Vertebral Column | 2 | 39 | 41 | 5% |
| <20 (juvenile) | PCG + Forelimb | 0 | 43 | 43 | 0% |
| <20 (juvenile) | PLG + Hindlimb | 2 | 26 | 28 | 7% |
| 21-42 (adult) | Skull | 1 | 31 | 32 | 3% |
| 21-42 (adult) | Ribs + Gastralia | 0 | 33 | 33 | 0% |
| 21-42 (adult) | Vertebral Column | 1 | 21 | 22 | 5% |
| 21-42 (adult) | PCG + Forelimb | 1 | 27 | 28 | 4% |
| 21-42 (adult) | PLG + Hindlimb | 1 | 12 | 13 | 8% |

*****PCG= Pectoral Girdle; PLG= Pelvic Girdle

**Coefficients:**

**Estimate Std. Error z value Pr(>|z|)**

(Intercept) -3.6434 0.7640 -4.769 1.85e-06 ***

Size21-42 0.2132 0.6015 0.354 0.723

RegionHindlimb 1.0326 0.9363 1.103 0.270

RegionRibs -0.0804 1.0144 -0.079 0.937

RegionSkull -0.1565 1.0133 -0.154 0.877

RegionVertebral Column 0.5685 0.9302 0.611 0.541

---

Signif. codes: 0 ‘***’ 0.001 ‘**’ 0.01 ‘*’ 0.05 ‘.’ 0.1 ‘ ’ 1

(Dispersion parameter for binomial family taken to be 1)

Null deviance: 2.55382 on 9 degrees of freedom

Residual deviance: 0.13547 on 4 degrees of freedom

AIC: 32.951

Number of Fisher Scoring iterations: 4

ANOVA

Analysis of Deviance Table

Model: binomial, link: logit

Response: cbind(Pathology, Not)

Terms added sequentially (first to last)

**Df Deviance Resid. Df Resid. Dev Pr(>Chi)**

NULL 9 2.55382

Size 1 0.05985 8 2.49397 0.8067

Region 4 2.35850 4 0.13547 0.6701

**Supplementary Table S2.** Logistic regression with aggregated data. Independent variables: Taxon and anatomical region (categorical predictors), pathological (binary response)

| **Taxon** | **Anatomical region** | | **Pathological** | **Not** | **Total** | **%** |
| --- | --- | --- | --- | --- | --- | --- |
| *Stenopterygius* | Skull | 6 | | 146 | 152 | 4% |
| *Stenopterygius* | Ribs + gastralia | 8 | | 132 | 140 | 6% |
| *Stenopterygius* | Vertebral Column | 2 | | 148 | 150 | 1% |
| *Stenopterygius* | PCG + Forelimb | 3 | | 142 | 145 | 2% |
| *Stenopterygius* | PLG + Hindlimb | 1 | | 125 | 126 | 1% |
| Mixosauridae | Skull | 2 | | 135 | 137 | 1% |
| Mixosauridae | Ribs + gastralia | 0 | | 134 | 134 | 0% |
| Mixosauridae | Vertebral Column | 3 | | 125 | 128 | 2% |
| Mixosauridae | PCG + Forelimb | 1 | | 123 | 124 | 1% |
| Mixosauridae | PLG + Hindlimb | 3 | | 51 | 54 | 6% |

**Coefficients:**

**Estimate Std. Error z value Pr(>|z|)**

(Intercept) -4.81218 1.00406 -4.793 1.65e-06 ***

TaxonStenopterygius 0.95497 1.16125 0.822 0.4109

RegionPelvic girdle + Hindlimb 1.97897 1.16665 1.696 0.0898 .

RegionRibs + gastralia -0.08566 1.41971 -0.060 0.9519

RegionSkull 0.60006 1.23107 0.487 0.6260

RegionVertebral Column 1.08248 1.16166 0.932 0.3514

TaxonStenopterygius:RegionPelvic girdle + Hindlimb -2.95007 1.64604 -1.792 0.0731 .

TaxonStenopterygius:RegionRibs + gastralia 1.13951 1.57751 0.722 0.4701

TaxonStenopterygius:RegionSkull 0.06531 1.42458 0.046 0.9634

TaxonStenopterygius:RegionVertebral Column -1.52933 1.48209 -1.032 0.3021

---

Signif. codes: 0 ‘***’ 0.001 ‘**’ 0.01 ‘*’ 0.05 ‘.’ 0.1 ‘ ’ 1

(Dispersion parameter for binomial family taken to be 1)

Null deviance: 1.5092e+01 on 9 degrees of freedom

Residual deviance: -1.1546e-14 on 0 degrees of freedom

AIC: 47.546

Number of Fisher Scoring iterations: 4

anova(glm.out, test="Chisq")

Analysis of Deviance Table

Model: binomial, link: logit

Response: cbind(Pathology, Not)

Terms added sequentially (first to last)

**Df Deviance Resid. Df Resid. Dev Pr(>Chi)**

NULL 9 15.092

Taxon 1 1.6665 8 13.425 0.19673

Region 4 2.6807 4 10.745 0.61260

Taxon:Region 4 10.7445 0 0.000 0.02959 *

---

Signif. codes: 0 ‘***’ 0.001 ‘**’ 0.01 ‘*’ 0.05 ‘.’ 0.1 ‘ ’ 1
